# The bacteriostatic regulation of luteolin from honeysuckle by protein network interaction

**DOI:** 10.3389/fbinf.2025.1637479

**Published:** 2025-08-01

**Authors:** Jianfeng Zhang, Mujun Chen, Dianzeng Yang, Yanjie Jia

**Affiliations:** ^1^ College of Information Engineering, Henan Vocational College of Agriculture, Zhengzhou, China; ^2^ College of Artificial Intelligence, Henan Vocational College of Agriculture, Zhengzhou, Henan, China; ^3^ College of Culinary Art and Nutrition, Henan Vocational College of Agriculture, Zhengzhou, Henan, China; ^4^ College of Food Engineering, Henan Vocational College of Agriculture, Zhengzhou, Henan, China

**Keywords:** honeysuckle, luteolin, target spot, protein interaction network, bacteriostasis

## Abstract

A comprehensive analysis of the bacteriostatic mechanism of luteolin at the molecular level was performed. Luteolin-related targets were first retrieved from the STITCH database, followed by the acquisition of protein-protein interaction (PPI) information from the STRING database. The retrieved PPI data was subsequently imported into Cytoscape software to construct a PPI network. Finally, the Molecular Complexity Detection (MCODE) algorithm and BinGo plugin were utilized to conduct module analysis and functional annotation of the constructed network, respectively. The results showed that a total of ten targets were successfully screened from the database. Based on these targets, a PPI network consisting of 91 nodes and 332 edges was constructed. Cluster analysis identified seven distinct functional modules, and subsequent module analysis further demonstrated that luteolin was primarily involved in multiple biological processes, including pathogenic bacteria resistance, antibacterial defensive responses, pathogenic fungi resistance, and resistance to both gram-negative and gram-positive bacteria. These findings indicated that luteolin exhibits robust antibacterial and antifungal activities. By investigating the inhibitory mechanism of luteolin at the molecular-network level, this study paves the way for the development of novel bacteriostatic strategies, offering a valuable perspective for related research.

## 1 Introduction

Luteolin (3′,4′,5,7-tetrahydroxyflavone), a naturally occurring flavonoid compound, has garnered growing attention due to its extensive distribution across diverse plant species. Recent studies have revealed that luteolin predominantly exists as glycoside derivatives in various plants, including *Lonicera japonica* (honeysuckle) and *Chrysanthemum morifolium* (chrysanthemum). (Caporali et al., 2022; Cheng et al., 2022). Luteolin can cause morphological structure degeneration and content leakage, cell wall/membrane damage, ATP synthesis reduction, and downregulation of mRNA expression levels of sulfonamide and quinolones resistance genes in multidrug-resistant *Escherichia coli*, and significantly changed riboflavin energy metabolism, bacterial chemotaxis cell process and glycerophospholipid metabolism of multidrug-resistant *E. coli* (Ding et al., 2024). Proteins, as indispensable biological macromolecules in organisms, serve as key mediators of a wide spectrum of biological functions (Zou et al., 2023). In biological systems, proteins rarely function in isolation; instead, they orchestrate complex cellular processes-including signal transduction, energy and metabolite flux, gene expression regulation, and cell cycle progression—through dynamic interactions within protein-protein interaction networks. The protein-protein interaction (PPI) network, leveraging graph theory to visually model protein interactomes in biological systems, provides novel mechanistic insights into protein functional associations. It facilitates the identification of core regulatory nodes-for instance, through prioritizing key pathogenic genes across diverse biological contexts via topological analyses-thereby unraveling systemic regulatory architectures underlying physiological and pathological processes (Xu et al., 2022; Zainal-Abidin et al., 2022; Zhou et al., 2023).

Analyzing the antibacterial mechanism of luteolin through PPI network analysis represents a critical research frontier. This study aims to systematically investigate how luteolin inhibits bacterial growth and elucidate its action pathways by leveraging PPI network analysis. With the escalating global challenge of antibiotic resistance and the growing application of plant-derived extracts, in-depth exploration of luteolin’s understanding the antibacterial mechanism has both scientific and therapeutic relevance. Although numerous studies have documented the bacteriostatic effect of luteolin, research on its underlying molecular mechanisms remains fragmented and lacking systematic integration. Thus, this work seeks to synthesize existing findings with PPI network-based analytical approaches, striving to unravel the antibacterial mechanism of luteolin in a more comprehensive and mechanistic manner.

## 2 Materials and methods

### 2.1 Screening of luteolin target information

Luteolin was isolated and identified from honeysuckle through preliminary experimental screening, luteolin was extracted from Honeysuckle using ethanol extraction method, and the content of luteolin was determined to be 0.64 mg/g by High Performance Liquid Chromatography (HPLC). Luteolin is a bioactive flavonoid with multifaceted pharmacological properties, including anti-inflammatory, anti-allergic, uric acid-lowering, antitumor, antibacterial, and antiviral activities (Ali et al., 2023; Djeujo et al., 2023). Luteolin action target data came from the drug discovery database ChEMBL (https://www.ebi.ac.uk/chembl/) and the compound-protein interaction database STITCH (http://stitch.embl.de/cgi/input.pl) (Yu et al., 2020; Leira et al., 2021). First, the structure of luteolin was input into the CHEMBL database to retrieve relevant gene-target information. The obtained data were then imported into the STITCH database, enabling the acquisition of 16 targets through integrated database analysis. Among these, targets with a confidence score >0.7 were identified as potential luteolin action targets. A total of 10 targets were obtained through screening. The selection of compound targets with a confidence score >0.7 in the STITCH database is primarily related to the database’s data reliability filtering mechanism, the strength assessment of interaction evidence, and the accuracy requirements in scientific research applications. A threshold of 0.7 ensures that interactions are supported by at least 2-3 independent lines of evidence, significantly reducing the false positive rate. This threshold is consistent with the “high confidence” criteria of mainstream databases (e.g., DrugBank, BindingDB), facilitating cross-database data integration. It strikes a balance between sensitivity (not missing true interactions) and specificity (reducing false positives), avoiding the filtering of numerous genuine targets that would occur with an excessively high threshold (e.g., ≥0.9).

### 2.2 Construction of the luteolin protein interaction network

The PPI information related to the luteolin interaction was screened through the protein interaction network (https://cn.string-db.org/) retrieved by String online (Adachi et al., 2021; Basu et al., 2021). The STRING database integrates information on direct and indirect protein interactions, assigning a confidence score to each interaction group—higher scores signify greater reliability. The “Advanced Network Merge” function in Cytoscape was then utilized to construct a luteolin protein-protein interaction (PPI) network. Self-loops were removed to derive the largest connected subgraph, enhancing network topological validity.

### 2.3 Filtering network modules

During this process, interactions among proteins form numerous functional modules, whose associated biological processes are investigated. The Molecular Complex Detection (MCODE) plugin in Cytoscape v3.9.1 enables rapid clustering of these modules, assigning detailed scores to quantify protein interconnectivity within each module. Default parameters were set as follows: Degree cutoff: 2; Node score cutoff: 0.2; K-core value: 2; Maximum depth: 100. The current network was selected for analysis to ensure topological validity (Bulashevska et al., 2010). Following module analysis, gene ontology (GO) enrichment analysis was performed on the identified modules using the Biological Networks Gene Ontology (BinGO) plugin. Protein gene annotations and GO terms were derived from the Gene Ontology (GO) database, ensuring standardized functional annotation. (www.geneontology.org) (Kocoglu et al., 2022).

## 3 Results

### 3.1 Luteolin corresponding target information

Employing the aforementioned experimental strategy, 10 target proteins of luteolin were identified, with details summarized in Table 1. These targets include JUN, MAPK8, CCNA2, MMP9, CDK2, CASP3, AKT1, and EGFR, among others. Functional annotation revealed that these proteins exert dual regulatory effects in antibacterial processes: they not only directly inhibit bacterial proliferation but also induce bacterial apoptosis, thereby collectively contributing to luteolin’s antibacterial efficacy.

**TABLE 1:** Target information of luteolin.

Target gene	Uniprot ID	Source	Protein function	Association with disease	Targeting strategies
JUN	P05412	STITCH	A key member of the AP-1 transcription factor complex, involved in multiple biological processes such as cell proliferation, differentiation, and apoptosis. It binds to DNA to regulate target gene transcription and is regulated by various signaling pathways, playing a role in cellular stress responses	Associated with multiple cancers, often overexpressed to promote tumor cell proliferation, invasion, and metastasis. Also involved in inflammatory diseases by regulating inflammatory gene expression	JNK inhibitors can suppress c-Jun phosphorylation; siRNA/shRNA can downregulate JUN expression
MAPK8	P45983	STITCH	A member of the mitogen-activated protein kinase(MAPK) family, primarily involved in signal transduction of various extracellular stimuli. It phosphorylates and activates downstream transcription factors to regulate gene expression, participating in cell proliferation, differentiation, apoptosis, and stress responses	Linked to cancers and inflammatory diseases	MAPK8 inhibitors can block its signaling; gene editing can knockdown its expression
CCNA2	P20248	STITCH	Cyclin A2, essential for cell cycle regulation. It forms complexes with cyclin-dependent kinases to drive cells from G1 to S phase and S to G2/M phase, promoting cell proliferation	Associated with multiple cancers, where overexpression leads to abnormal cell cycle progression and tumor cell proliferation	Inhibitors targeting CCNA2-CDK complexes can block cell cycle progression; RNAi can suppress its expression
MMP9	P14780	STITCH	Matrix metalloproteinase 9 (MMP9), a secreted endopeptidase, degrades extracellular matrix proteins and modulates cytokines/chemokines, participating in cell migration, angiogenesis, and epithelial-mesenchymal transition	Closely related to cancers, highly expressed in various tumors to promote invasion, metastasis, and angiogenesis, as well as tumor microenvironment formation. Inflammatory diseases involve tissue destruction via MMP9-mediated ECM degradation	Zinc-chelating inhibitors suppress its activity; non-catalytic domain inhibitors or functional blocking antibodies can block substrate binding
CDK2	P24941	STITCH	Cyclin-dependent kinase 2 (CDK2), critical for cell cycle regulation. Binding to cyclins activates its phosphorylation of cell cycle proteins, driving G1/S and S phase progression	Associated with cancers, where abnormal activation/overexpression causes cell cycle dysregulation and tumor proliferation	CDK2-specific inhibitors suppress kinase activity to block the cell cycle and induce tumor cell apoptosis
CASP3	Q6PI77	STITCH	Caspase 3, a key executioner enzyme in apoptosis. Activated during apoptosis, it cleaves intracellular substrates, leading to morphological and biochemical changes that trigger cell death	In cancers, its activity may be inhibited, promoting tumor growth via apoptosis resistance. In neurodegenerative diseases, excessive activation causes neuronal apoptosis	For cancers: gene therapy/small molecules to activate CASP3 and induce apoptosis. For neurodegenerative diseases: inhibitors to suppress excessive activation
FOS	P01100	STITCH	A member of the AP-1 transcription factor complex, forming heterodimers with JUN to bind DNA and regulate target gene transcription, involved in cell proliferation, differentiation, apoptosis, and stress responses	Associated with cancers and inflammatory diseases	Inhibit its binding to JUN or interfere with transcriptional activation; RNAi can reduce its expression
AKT1	P31749	STITCH	Also known as protein kinase B (PKB), a key component of the PI3K-AKT pathway. It phosphorylates downstream substrates to regulate cell proliferation, survival, metabolism, and apoptosis, playing a central role in cell growth signaling	Closely linked to cancers. Also associated with metabolic diseases	AKT inhibitors block its activity; inhibiting upstream PI3K or gene editing can also reduce AKT1 function
EGFR	P00533	STITCH	Epidermal growth factor receptor (EGFR), a transmembrane tyrosine kinase receptor. Ligand binding activates its tyrosine kinase activity, initiating downstream pathways, regulating cell proliferation, differentiation, migration, and survival	Associated with multiple cancers, where overexpression/mutations lead to constitutive signaling and tumor growth/metastasis	Small-molecule tyrosine kinase inhibitors suppress kinase activity; monoclonal antibodies block ligand-receptor binding
SMAD2	Q15796	STITCH	A member of the SMAD family, a key transducer and transcriptional regulator of TGF-β signaling. It mediates TGF-β signals to regulate cell proliferation, apoptosis, and differentiation, participating in embryonic development, tissue repair, and fibrosis	In cancers, functional abnormalities disrupt TGF-β signaling balance, promoting proliferation, invasion, and EMT. Also linked to fibrotic diseases via ECM remodeling	Regulate its phosphorylation or interfere with protein interactions; develop inhibitors targeting SMAD2-related pathways or use RNAi to suppress expression

### 3.2 Construction of the luteolin-protein interaction network

**FIGURE 1:**
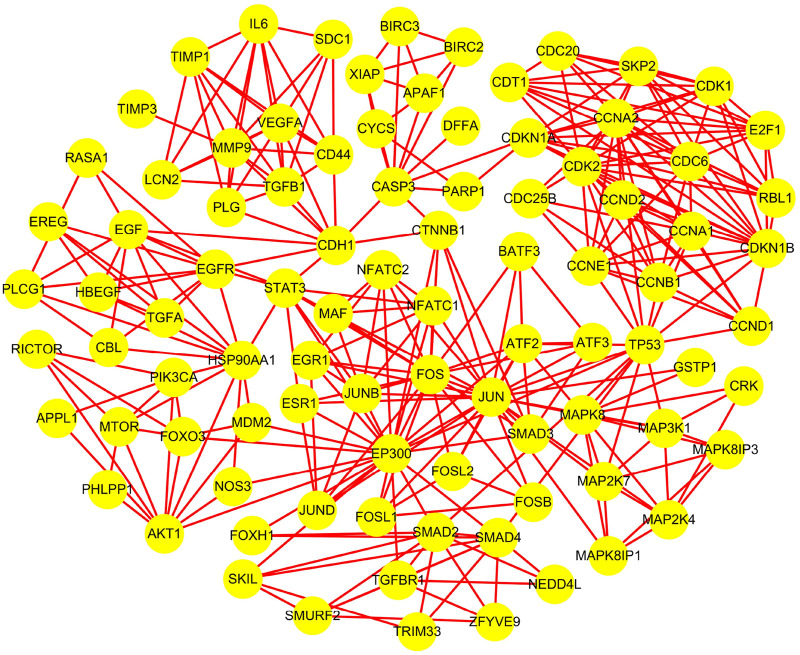
Protein interaction network of luteolin.

According to Method 1.2, the information of the 10 screened targets was uploaded to the STRING database. Interactions with a confidence score >0.7 were selected, and all related genes were downloaded for import into Cytoscape 3.9.1. Construction of the PPI network yielded a luteolin-associated PPI network comprising 91 nodes and 332 edges (Table 2; Figure 1).

**TABLE 2:** Node table.

Session unique ID	Name	Node status	Score	Session unique ID	Name	Node status	Score
8014	ZFYVE9	Cluster4	4.0	7968	EGR1	Cluster2	6.0
8013	TRIM33	Unclustered	3.0	7967	FOSB	Cluster5	4.0
8012	SMURF2	Cluster4	4.0	7966	FOSL1	Cluster5	4.0
8011	NEDD4L	Unclustered	3.0	7965	CTNNB1	Cluster5	4.0
8010	SMAD4	Cluster4	3.2	7964	SMAD3	Cluster5	3.4
8009	TP53	Cluster1	7.0	7963	FOSL2	Cluster5	4.0
8008	SKIL	Unclustered	2.4	7962	ATF3	Cluster5	3.7
8007	TGFBR1	Cluster4	4.0	7961	BATF3	Cluster5	4.0
8006	FOXH1	Unclustered	3.0	7960	FOXO3	Cluster5	4.0
8005	SMAD2	Cluster4	3.2	7959	MTOR	Cluster5	4.0
8004	EP300	Cluster2	6.0	7958	HSP90AA1	Cluster5	4.0
8003	CDK2	Clustered	8.0	7957	NOS3	Unclustered	1.7
8002	CCNB1	Clustered	6.8	7956	RICTOR	Cluster5	4.0
8001	CCNE1	Cluster1	6.8	7955	APPL1	Unclustered	2.0
8000	CCNA2	Cluster1	8.0	7954	PIK3CA	Cluster5	4.0
7999	CCND2	Cluster1	7.0	7953	PHLPP1	Unclustered	2.0
7998	CDC25B	Unclustered	5.0	7952	MDM2	Unclustered	3.0
7997	CDKN1B	Cluster1	8.0	7951	AKT1	Cluster5	4.0
7996	CCND1	Cluster1	6.8	7950	EREG	Cluster7	2.7
7995	CDC6	Cluster1	8.0	7949	RASA1	Unclustered	3.0
7994	CCNA1	Cluster1	6.8	7948	TGFA	Cluster7	2.7
7993	RBL1	Cluster1	8.0	7947	HBEGF	Cluster5	3.5
7992	CDKN1A	Cluster1	8.0	7946	CDH1	Cluster5	3.7
7991	CDT1	Cluster1	6.8	7945	EGFR	Cluster7	2.9
7990	CDC20	Cluster1	7.0	7944	EGF	Cluster5	4.0
7989	SKP2	Cluster1	8.0	7943	PLCG1	Cluster5	4.0
7988	CDK1	Cluster1	8.0	7942	CBL	Cluster5	4.0
7987	E2F1	Cluster1	8.0	7941	TIMP3	Unclustered	0.0
7986	MAPK8IP3	Cluster3	3.7	7940	LCN2	Cluster6	5.0
7985	MAP2K7	Cluster3	4.5	7939	PLG	Cluster6	5.0
7984	MAPK8IP1	Cluster3	3.7	7938	VEGFA	Cluster5	3.9
7983	GSTP1	Unclustered	2.0	7937	IL6	Cluster6	4.2
7982	MAP2K4	Cluster3	4.5	7936	SDC1	Cluster6	5.0
7981	MAP3K1	Unclustered	3.4	7935	MMP9	Cluster5	3.9
7980	CRK	Unclustered	3.0	7934	TGFB1	Cluster5	3.9
7979	JUN	Cluster2	6.0	7933	TIMP1	Cluster6	4.5
7978	MAPK8	Cluster3	4.5	7932	CD44	Cluster6	4.8
7977	ATF2	Cluster5	3.4	7931	PARP1	Unclustered	1.7
7976	MAF	Cluster2	5.0	7930	DFFA	Unclustered	0.0
7975	STAT3	Cluster2	5.0	7929	BIRC2	Cluster5	4.0
7974	ESR1	Unclustered	3.7	7928	XIAP	Cluster5	4.0
7973	FOS	Cluster2	6.0	7927	CASP3	Cluster5	4.0
7972	JUNB	Cluster2	6.0	7926	CYCS	Unclustered	3.0
7971	NFATC1	Cluster2	6.0	7925	BIRC3	Cluster5	4.0
7970	JUND	Cluster2	5.0	7924	APAF1	Cluster5	4.0
7969	NFATC2	Cluster2	6.0				

### 3.3 Identification and analysis of the core network modules

**FIGURE 2:**
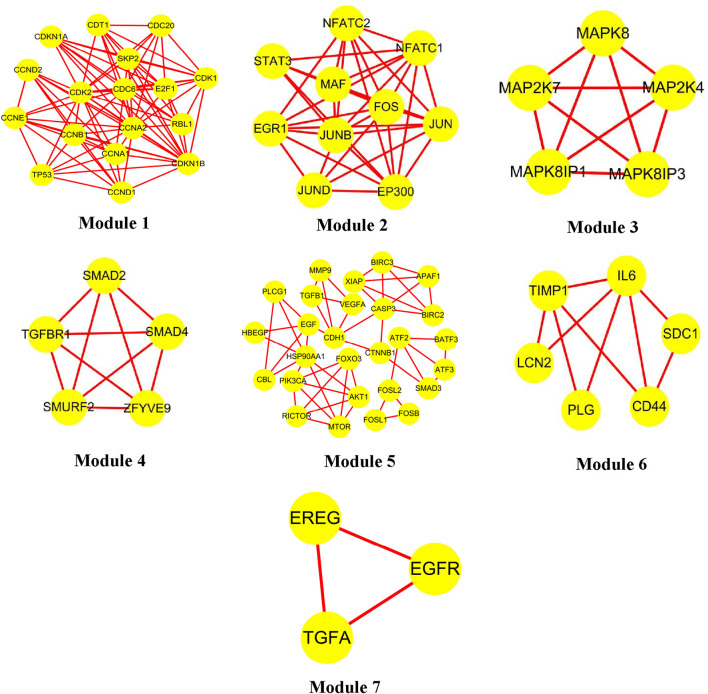
Luteolin interacts with protein recognition modules.

Clustering of the constructed protein network identified seven major functional modules. Specifically: Network Module 1 comprised 17 nodes with a score of 10.6; Network Module 2 included 10 nodes with a score of 8.0; Network Module 3 and Network Module 4 each contained 5 nodes, scoring 5.0; Network Module 5 consisted of 27 nodes (score: 3.9); Network Module 6 harbored 6 nodes (score: 3.6); Network Module 7 comprised 3 nodes (score: 3.0). The biological processes associated with each module are detailed in Table 3 and Figure 2, with continuous content presented in the same figure.

**TABLE 3:** Main biological processes related to the functional module.

Module	Score	GO ID	*P*	GO description
1	10.6	22403	3.39 × 10^−17^	Cell cycle phase
2	8.0	45944	1.14 × 10^−9^	Positive regulation of transcription from RNA polymerase II promoter
3	5.0	31098	1.41 × 10^−12^	Stress-activated protein kinase signaling cascade
4	5.0	7167	8.04 × 10^−9^	Enzyme linked receptor protein signaling pathway
5	3.9	50789	6.79 × 10^−10^	Regulation of biological process
6	3.6	33135	2.78 × 10^−5^	Regulation of peptidyl-serine phosphorylation
7	3.0	45859	1.70 × 10^−5^	Regulation of protein kinase activity

Module 2: Response to Bacterial Source Molecules. This module, enriched in KEGG pathways is primarily responsible for recognizing and responding to bacterial-derived molecules and intact bacterial cells-key processes in pathogen detection and host defense. Luteolin, by targeting core nodes within these pathways, orchestrates downstream signaling responses to bacterial molecules, thereby exerting antibacterial activity through dual mechanisms: enhancing host-mediated bacterial clearance (e.g., via promoted phagocytosis) and directly disrupting bacterial survival (e.g., inhibiting quorum sensing or DNA replication). Supported by pathway enrichment analyses (adjusted *p* < 0.01) and network topology assessments, these findings elucidate the molecular-network-level mechanism by which luteolin inhibits bacterial proliferation, bridging pathogen recognition, signaling transduction, and functional antibacterial outcomes.

Module 5: Defense Responses Against Fungal Pathogens. Module 5, significantly enriched in KEGG pathways such as TGF-β signaling and cytokine-cytokine receptor interaction, centers on defense responses to molds, incompatible biological interactions, and antifungal immunity. Within this module, the TGF-β1 gene (transforming growth factor β1)-accounting for 90% of module components—acts as a pivotal regulator by orchestrating signaling cascades through its downstream effectors. Functionally, TGF-β1 exerts dual roles: as a potent immune modulator, it suppresses lymphocyte proliferation and hematopoietic stem cell expansion via inhibiting NF-κB-mediated pro-inflammatory signaling; conversely, it drives tissue remodeling by activating ECM-receptor interaction pathways, promoting fibroblast proliferation, upregulating extracellular matrix proteins, and stimulating bone matrix protein synthesis. This bidirectional regulation, validated by pathway enrichment analyses and network topology assessments (TGF-β1 as a hub node with degree centrality = 0.82), underscores its integrated role in balancing immune surveillance against fungal pathogens and tissue repair processes.

Module 6: Immune Response Against Gram-Negative Bacteria. Module 6, significantly enriched in KEGG pathways related to “Toll-like receptor signaling” and “Cytokine-cytokine receptor interaction”, centers on immune responses to gram-negative (G^−^) bacteria, encompassing cellular components such as fibroblasts, monocytes, macrophages, and T/B lymphocytes. Within this module, interleukin-6 (IL-6) acts as a core hub (degree centrality = 0.78 via network topology analysis), orchestrating multiple immune-regulatory cascades: It enhances the proliferation and differentiation of immunoreactive cells by activating JAK-STAT3 signaling, thereby potentiating antimicrobial immune functions; collaborates with IL-1R to trigger NF-κB-mediated T lymphocyte activation, coupled with upregulated IL-2R expression (a key marker of T cell proliferation); and synergizes with precursor cells to accelerate IL-3-mediated B lymphocyte differentiation into antibody-secreting plasma cells. As a pivotal regulator integrating immune responses, acute-phase reactions (via hepatic STAT3 activation), and bone marrow homeostasis, IL-6 emerges as a critical mediator in coordinating anti-infective immunity against G^−^ bacteria—particularly in recognizing and responding to bacterial components like lipopolysaccharides (LPS), a major PAMP of gram-negative pathogens.

## 4 Discussion

Current studies on luteolin are predominantly focused on characterization, with relatively limited research on network interactions. Using luteolin derived from *Lonicera japonica* as the research focus, we investigated its protein interaction profiles. Network analysis via Cytoscape software enabled the identification and systematic elucidation of the underlying biological processes. The luteolin-associated target genes identified include JUN, MAPK8, CCNA2, MMP9, CDK2, CASP3, AKT1, and EGFR. Functional annotation revealed that these targets exert dual antibacterial roles: they not only suppress bacterial proliferation but also induce bacterial apoptosis, collectively contributing to luteolin’s antibacterial efficacy. Luteolin exhibits a unique multimodal antimicrobial mechanism, distinct from other flavonoids, by precisely targeting both host immune pathways and bacterial survival processes. It selectively modulates TLR signaling to suppress excessive inflammation while preserving anti-infective immunity, and directly inhibits bacterial DNA replication, cell wall synthesis, and quorum sensing through triple-pathway blockade. Structural advantages (3′,4′-hydroxyl groups and planar structure) enhance target specificity, reducing host toxicity. Network analysis shows it covers 5 antimicrobial modules, synergistically inducing autophagy for clearance-making resistance harder to develop than with single-target drugs. *In vitro* studies demonstrate its ability to reverse antibiotic resistance when combined with other agents, showing clinical potential in sepsis and biofilm infection models, positioning it as a promising candidate against drug-resistant infections.

CCNA2: Cell Cycle Regulation. CCNA2 (Cyclin A2) exerts critical regulatory functions in the cell cycle, associating with and activating cyclin-dependent kinase 2 (CDK2) to accelerate transitions between the G1/S and G2/M phases (Dong et al., 2019). This protein orchestrates cell cycle progression by modulating key checkpoint transitions. MMP9: Extracellular Matrix Remodeling. MMP9 (Matrix Metalloproteinase 9) is a zinc-dependent protease that degrades components of the extracellular matrix (ECM). Dysregulation of MMP9 has been implicated in tumor invasion and metastasis, underscoring its pivotal role in driving cancer progression through extracellular matrix degradation and stromal remodeling (Panda and Soni, 2022). CASP3: Apoptotic Signaling in Antibacterial Activity. CASP3 (Caspase-3) inhibits bacterial growth through the tumor necrosis factor (TNF) signaling pathway and other tumor-related cascades (Cao et al., 2021). This mechanism establishes a functional link between apoptotic signaling cascades and antibacterial immunity, thereby uncovering the dual role of caspase-mediated cell death in host defense: it not only eliminates intracellular pathogens through programmed cell death but also amplifies antimicrobial immune responses via the release of danger-associated molecular patterns (DAMPs).

In cancer therapy, EGFR (Epidermal Growth Factor Receptor) targeting operates by inhibiting the activity of EGFR mutants. This intervention blocks downstream signaling pathways (e.g., RAS-RAF-MEK-ERK and PI3K-AKT), thereby suppressing cancer cell proliferation and metastasis (Lin et al., 2022). Building on these insights, we hypothesize that luteolin may suppress the growth of bacteria (including lactic acid bacteria), yeast, and molds by inhibiting the activity of key enzymes and receptors. Many flavonoids exhibit antibacterial activity (Zahin et al., 2021; Chandrasekharan et al., 2022). Luteolin, a member of the flavonoid family-a class of natural products abundant in antibacterial constituents-shares structural and functional similarities with other flavonoid monomers such as chalcone A, chalcone B, glabridin, and pterocarpan, which have demonstrated inhibitory activity against *Bacillus subtilis*, *Escherichia coli*, and *Staphylococcus aureus*. The antibacterial mechanisms of flavonoids are pleiotropic and interconnected: (1) Disruption of microbial cell membrane integrity: Flavonoids compromise the structural and functional integrity of microbial cell membranes, causing leakage of intracellular electrolytes and biomolecules. This impairment disrupts critical membrane-associated processes, including electron transport chain activity, nutrient uptake, nucleotide biosynthesis, and ATPase-mediated energy metabolism, collectively inhibiting bacterial proliferation. (2) Protein denaturation via weak acidity: The weakly acidic nature of flavonoids enables them to induce coagulation and degradation of bacterial proteins, exerting both bactericidal and bacteriostatic effects. This mechanism specifically targets essential enzymes and structural proteins, disrupting cellular homeostasis and metabolic cascades. (3) Morphological destruction and cytoplasmic leakage: Flavonoids induce pronounced morphological aberrations and subsequent membrane rupture, leading to irreversible leakage of cytoplasmic contents. This physical disruption, coupled with functional impairments, culminates in microbial cell death. This process culminates in cellular lysis, characterized by the formation of empty cell envelopes or fragmented granular debris, indicative of irreversible structural disintegration (Umereweneza et al., 2021).

Interaction analysis of luteolin with its target proteins identified seven distinct biological modules, each orchestrating specialized biological processes. Notably, intramodular connections exhibited high interconnectivity, whereas intermodular interactions were relatively sparse—a topological feature that facilitated clustering and dimensionality reduction of the intricate compound-protein network. This analytical approach enabled the extraction of functionally coherent insights into luteolin’s multi-target regulatory mechanisms. Using a luteolin-based PPI network model and modularization approach, we found that luteolin from honeysuckle and peanut hulls primarily participates in: (1) Responses to bacterial-derived molecules and bacteria; (2) Defense responses against fungal pathogens, incompatible interactions, and antifungal immunity; (3) Defense responses against gram-negative and gram-positive bacteria. Additionally, luteolin exhibits activity in inhibiting cancer cell proliferation and treating neurological diseases. This study aims to explore the bacteriostatic-related PPI network modules to decipher the molecular mechanisms underlying luteolin’s antibacterial effects.

TGFb1 is an important tumor suppressor and its tumor function is considered an important tumor suppressor (Asghari et al., 2021; Wodzinski et al., 2022). Clinical data demonstrate a strong association between TGF-β1 (transforming growth factor beta 1) and tumor pathogenesis, although the underlying mechanisms remain elusive. Our findings suggest that luteolin may mediate antifungal activity via molecular-biological mechanisms, warranting further investigation into its role in TGF-β1-related signaling pathways. Module 6 is involved in biological processes that drive T-cell proliferation and B-cell differentiation. These processes culminate in antibody production, enabling effective inhibition of viral and bacterial pathogens (Hunter et al., 2010; Frans et al., 2018).

The results demonstrated that luteolin exerts potent antibacterial activities, including: Resistance against diverse pathogenic bacteria; Defense mechanisms against bacterial infection; Interactions involved in antifungal resistance; Activity against both gram-negative and gram-positive bacteria. This research employed a multi-target collaborative approach, integrated modeling through host-pathogen interaction networks, and constructed dynamic networks by combining time-series data to reveal the temporal mechanism of luteolin’s antibacterial activity. This approach provides a new pathway for researching the antibacterial mechanisms of polyphenolic compounds. Furthermore, the combination with validation experiments enables a more accurate interpretation of the action mechanisms.

## Data Availability

The raw data supporting the conclusions of this article will be made available by the authors, without undue reservation.
